# Release of Hypoglycin A from Hypoglycin B and Decrease of Hypoglycin A and Methylene Cyclopropyl Glycine Concentrations in Ruminal Fluid Batch Cultures

**DOI:** 10.3390/toxins17020046

**Published:** 2025-01-21

**Authors:** Anna Maria Engel, Ahmed H. El-Khatib, Martin Bachmann, Monika Wensch-Dorendorf, Fenja Klevenhusen, Stefan Weigel, Robert Pieper, Annette Zeyner

**Affiliations:** 1 German Federal Institute for Risk Assessment, 10589 Berlin, Germanyahmed.el-khatib@bfr.bund.de (A.H.E.-K.); stefan.weigel@bfr.bund.de (S.W.); robert.pieper@bfr.bund.de (R.P.); 2 Institute of Agricultural and Nutritional Sciences, Martin Luther University Halle-Wittenberg, 06120 Halle (Saale), Germany; monika.dorendorf@landw.uni-halle.de (M.W.-D.); annette.zeyner@landw.uni-halle.de (A.Z.); 3 Faculty of Organic Agriculture, University of Kassel, 37213 Witzenhausen, Germany; fenja.klevenhusen@uni-kassel.de

**Keywords:** sycamore maple tree, secondary plant metabolites, phytotoxins, rumen fermentation, atypical myopathy

## Abstract

The transformation of hypoglycin A (HGA), hypoglycin B (HGB), and methylene cyclopropyl glycine (MCPrG) in ruminal fluid batch cultures was investigated, and the effect of these toxins on the batch culture microorganisms using microbial metabolites was measured. An experiment was conducted using ovine ruminal fluid batch cultures and the ANKOM RF Gas Production System over four runs, each with an incubation period of 48 h. The fermenters contained 200 mg of (i) a substrate mixture (80% cellulose, 20% starch; CSM), (ii) CSM and 1.5 mL of a solution of pure toxins (a mixture of 500 ng/mL HGA and MCPrG each; PCM), or (iii) CSM and 100 mg sycamore maple seeds (SMS). Each fermenter contained 30 mL of inoculum (ruminal fluid and buffer, 1:2 *v*/*v*). For control, autoclaved ruminal fluid was incubated with CSM, PCM, and SMS, respectively. Samples were taken from the liquid phase of the fermenters and analyzed using liquid chromatography–tandem mass spectrometry (LC/MS-MS) for sycamore maple toxins and metabolites. Microbial activity was assessed using gas production, short chain fatty acids, and NH_3_ concentration. Additionally, pH and redox potentials were measured. In PCM, HGA and MCPrG concentrations rapidly decreased (*p* < 0.05), and were not measurable anymore after a 24 h incubation period. In SMS, the initial concentrations were 4.7 ± 1.4 µg/mL HGA, 19.9 ± 5.41 µg/mL HGB, and 1.2 ± 0.33 µg/mL MCPrG. In SMS, HGA increased in 24 h, coincidently to a decrease in HGB concentration (*p* < 0.05). We modeled a rapid conversion of HGB to HGA, accompanied by progressive HGA transformation. The concentration of MCPrG was constant until 4 h and decreased afterwards (*p* < 0.05). In SMS incubations, HGA and MCPrG concentrations of 5.6 ± 1.5 and 0.32 ± 0.090 µg/mL remained after 48 h, respectively. The HGB to HGA conversion and transformation of HGA and MCPrG also occurred in autoclaved ruminal fluid. Gas production and microbial metabolite concentrations were higher in SMS compared to CSM and PCM (*p* < 0.05), as the seeds were used as an additional substrate by the batch culture microorganisms.

## 1. Introduction

**Figure 1:**
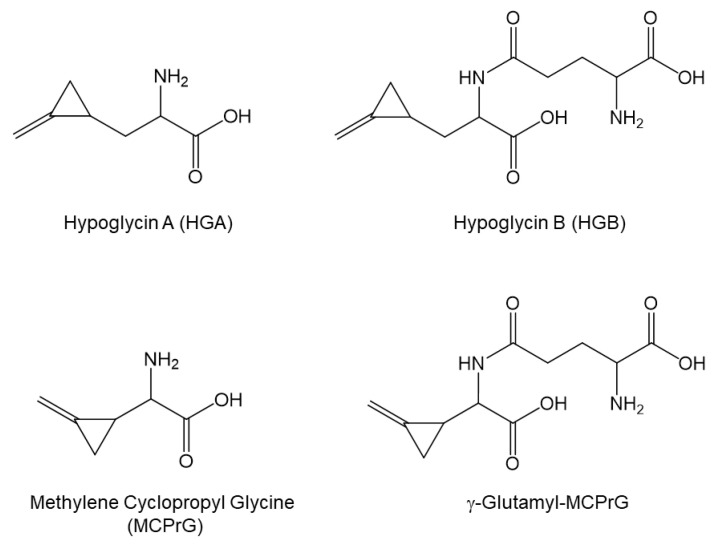
Chemical structures of the toxins investigated in this study.

The toxins hypoglycin A (HGA) and methylene cyclopropyl glycine (MCPrG), and their glutamyl derivatives hypoglycin B (HGB) and γ-glutamyl-MCPrG (Figure 1), are non-proteinogenic amino acids that occur as secondary plant metabolites in plants of the soap tree family (*Sapindaceae*). The sycamore maple tree (*Acer pseudoplatanus*) is a deciduous tree of the *Sapindaceae* native to Central and Eastern Europe, which has a high spreading potential [1,2,3,4,5]. The *Sapindaceae* family also includes other maple species, native in other areas of the world, that may pose a potential risk to herbivore animals [6]. The ingestion of maple seeds and seedlings regularly causes severe poisoning, known as seasonal pasture or atypical myopathy. Cases have been reported in horses, Père David’s deer, gnus, and Bactrian camels [2,7,8,9,10]. Following ingestion and absorption, HGA and MCPrG are metabolized in the liver into their coenzyme A adducts methylene cyclopropyl acetyl-CoA (MCPA-CoA) and methylene cyclopropyl formyl-CoA (MCPF-CoA), which trigger the clinical manifestation of poisoning. Affected individuals show rhabdomyolysis with finely dispersed intracellular lipid droplets in affected skeletal muscles, typical of lipid-accumulation myopathy [11,12]. This follows inhibition of β-oxidation of fatty acids [13]. In addition, MCPA-CoA and MCPF-CoA lead to a depletion in hepatic adenosine triphosphate (ATP) stores [13]. In this way, they reduce the energy available for gluconeogenesis [13]. An accumulation of acyl conjugates in blood and urine is indicative [12,14]. In contrast to HGA and MCPrG, little is known about the metabolism of HGB and γ-glutamyl-MCPrG. Hypoglycin B has been detected in maple seeds and seedlings [15,16]. In ackees (*Blighia sapida*), HGB is considered a reservoir for HGA and its portion increases during the ripening of the fruit [17]. An inverse relationship between the concentration of HGA in arilli and seeds and HGB in seeds has been reported [17]. The HGA to HGB conversion is catalyzed by γ-glutamyl-transpeptidase [17,18]. The mechanisms and kinetics of HGA release from HGB in biological environments are largely unknown. The in vitro incubation of milled maple seeds in ruminal fluid led to an increase in HGA concentration within 2 h [19]. This HGA could originate from HGB and this observation scrutinizes the possible role of HGB in maple poisoning events [16]. Ruminants such as cattle and sheep seem to be less susceptible to intoxication [10,20]. An explanation for this could be that parts of the toxins HGA and MCPrG are not metabolized into the toxic conjugates MCPA-CoA and MCPF-CoA, respectively, but transformed in the rumen. However, the metabolites MCPA-glycine, MCPA-carnitine, MCPF-glycine, and MCPF-carnitine were found in the milk of grazing dairy cows, which still indicates a significant transfer across the blood–udder barrier [12,20,21]. The interaction of maple toxins and metabolites with the microbial community in the rumen is still unknown.

In the present in vitro experiment, a mixture of pure sycamore maple toxins (HGA and MCPrG) and sycamore maple seeds (SMS) were incubated in a ruminal fluid batch culture over a period of 48 h, considering forestomach mean retention times that usually range from 15 to 35 h [22]. The objectives of the study were to investigate the changes in the concentration of HGA, HGB, and MCPrG in the batch cultures, and the effect of the toxins on the batch culture microorganisms using microbial metabolites, i.e., short chain fatty acids (SCFAs) and NH_3_, as measurands. We hypothesized that (i) during the time of incubation, HGA is released from HGB; (ii) HGA and MCPrG are subjected to continuous transformation; and (iii) the presence of the toxins affects microbial metabolic profiles.

## 2. Results and Discussion

### 2.1. Fate of Maple Toxins

**Figure 2:**
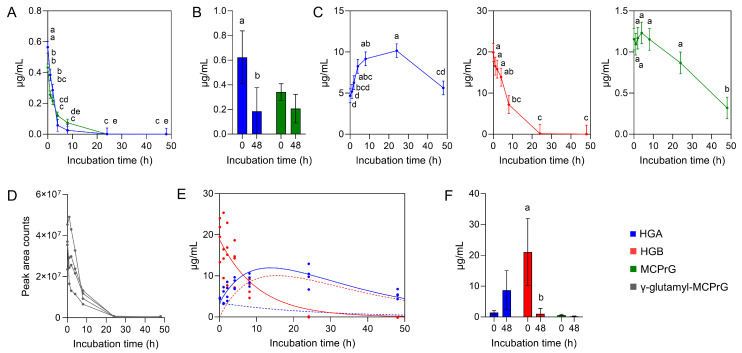
Changes in the concentration of sycamore maple toxins during 48 h of incubation in a ruminal fluid batch culture. Concentrations of HGA and MCPrG from PCM (**A**). Concentrations of HGA and MCPrG from PCM incubated in autoclaved ruminal fluid/buffer solution (**B**). Concentrations of HGA, HGB, and MCPrG from SMS (**C**). Peak area counts associated with γ-glutamyl-MCPrG shown for four individual runs of SMS incubation (**D**). Estimated time-dependent change in HGA and HGB concentration (**E**). Solid lines represent predicted concentration changes. Dotted lines represent the possible time-dependent reduction in HGA_0_ (HGA concentration at time point *t* = 0 h) and conversion of HGB to HGA (both not measurable). Blue lines represent changes in HGA concentration, red lines represent changes in HGB concentration. Dots represent the individual measurements, i.e., four consecutive runs. The predicted maximal HGA concentration was reached after 13.7 h. Concentrations of HGA, HGB, and MCPrG from SMS incubated in autoclaved ruminal fluid/buffer solution (**F**). All substrates were incubated together with a mixture of 80% cellulose and 20% starch. Incubations were carried out in vital ruminal fluid/buffer solution unless otherwise stated. Values are given as means and standard deviation. Abbreviations: HGA, hypoglycin A; HGB, hypoglycin B; MCPrG, methylene cyclopropyl glycine; PCM, pure chemical mixture (HGA and MCPrG); SMS, sycamore maple seeds. ^a–e^ Superscripts indicate differences among incubation time points with *p* < 0.05.

Incubation of pure HGA/MCPrG mixture (PCM): Over the course of the incubation of PCM in ruminal fluid batch cultures, HGA and MCPrG concentrations decreased (Figure 2A). From initially 564 ± 133 ng/mL HGA, only 25.3 ± 32.2 ng/mL HGA was detected after 8 h (*p* < 0.05). After 24 h of incubation, no HGA was detected. From initially 432 ± 85.1 ng/mL MCPrG, only 74.4 ± 53.1 ng/mL MCPrG was detected after 8 h (*p* < 0.05), and nothing after 24 h. The decrease in the concentration of MCPrG was slower than the decrease in the concentration of HGA (mean t1/2 of 2.20 and 2.05 h, respectively). No HGB was detected in samples collected from PCM incubations. The metabolites MCPA-glycine, MCPA-carnitine, and MCPF-glycine were not detected either.

The incubation of PCM in an inoculum made from autoclaved ruminal fluid resulted in a reduction in HGA and MCPrG concentrations as well (Figure 2B). In contrast to PCM incubated in vital ruminal fluid, the toxins were not fully cleared. Initial concentrations were 587 ± 198 ng/mL HGA and 326 ± 74.3 ng/mL MCPrG. Concentrations of 186 ± 192 and 207 ± 116 ng/mL were measured after 48 h for HGA and MCPrG, respectively.

A possible explanation for the decrease in the HGA and MCPrG concentrations in the active ruminal fluid is the activity of microbial enzymes in this medium. As pure substances in solution, HGA and MCPrG are immediately accessible to the enzymes in the batch culture. Amino acids are incorporated into microbial protein [23]. Amino acids with an excess of microbial needs can be deaminated to NH_3_ [24,25]. Non-proteinogenic amino acids such as the maple toxins often serve as a structural mimicry to proteinogenic amino acids, and may therefore be involved in the same metabolic pathways [26]. Another conceivable explanation would be that the toxins bind to matrix components (see below) and are transported in this form to the place of metabolization, e.g., the liver.

The hypothesis of biological transformation by rumen microorganisms is challenged by the observation that in the incubations carried out with autoclaved ruminal fluid, a decrease in the HGA and MCPrG concentrations occurred as well, although this was not as strong as in the incubations with active ruminal fluid. No considerable gas production was measured in the fermenters inoculated with autoclaved ruminal fluid, which is why we assume that no biological activity was present. Thus, it is likely that the observed decrease in HGA and MCPrG concentrations was at least partly the result of an abiotic process. As HGA is stable in pure water, which we tested, the decrease must be related to the components and conditions (pH and redox potential) present in the incubation fluid. A hypothetic adsorption or binding to proteins, amino acids, or fatty acids would have to be verified by additional experiments.

Incubation of SMS: In SMS incubations carried out with active ruminal fluid, 100 mg ground seeds were used and the initial HGA concentration was 4.7 ± 1.4 µg/mL. The initial concentration of MCPrG was approximately 4-fold lower than that of HGA, 1.2 ± 0.33 µg/mL. There was also a high initial concentration of HGB, 19.9 ± 5.41 µg/mL (Figure 2C). In SMS incubations, HGA increased in 24 h (*p* < 0.05), which was accompanied by a coincident decrease in HGB concentration (*p* < 0.05) (Figure 2C). The decrease in HGA concentration became measurable after the maximal concentration of HGA was reached, and was measured at 24 h of incubation (Figure 2C). For γ-glutamyl-MCPrG, the HGB analog of MCPrG, unequivocal confirmation and quantification was not possible, due to the lack of a reference standard. However, the presence of γ-glutamyl-MCPrG in sycamore maple seeds was already demonstrated using high-resolution mass spectrometry (hrMS) [16]. Within a mass tolerance of 3 ppm, we detected the accurate mass of γ-glutamyl-MCPrG. Two probable mass transitions for γ-glutamyl-MCPrG have been monitored, for which the chromatographic peak areas were used to investigate the trends in levels of γ-glutamyl-MCPrG over the 48 h run. The results are shown in Figure 2D. On the basis of the measured concentration changes, we applied a model to simulate HGA and HGB concentration kinetics. This model predicted that HGA concentration started to decrease after 13.7 h of the incubation period (Figure 2E). In SMS incubations, high HGA and MCPrG concentrations (5.6 ± 1.5 and 0.32 ± 0.090 µg/mL, respectively) remained after 48 h (Figure 2C). Again, MCPA-glycine, MCPA-carnitine, and MCPF-glycine have not been detected.

The incubation of SMS in autoclaved ruminal fluid led to an increase in HGA and decrease in HGB concentrations (Figure 2F). It has to be emphasized that the sonication of solutions of standard HGB in water and in water containing 1% formic acid (pH 2.7), followed by liquid chromatography–tandem MS (LC-MS/MS) measurement, showed no signal for HGA. This indicates the stability of HGB in aqueous solutions. However, in contrast to unautoclaved ruminal fluid, HGB was not fully cleared after 48 h (Figure 2F). Initial concentrations were 1.4 ± 0.64 µg/mL HGA, 21.1 ± 11.0 µg/mL HGB, and 0.64 ± 0.24 µg/mL MCPrG. After 48 h, the remaining concentrations were 8.7 ± 6.6, 1.0 ± 1.2, and 1.2 ± 1.5 µg/mL HGA, HGB, and MCPrG, respectively.

In the SMS incubations, the concentration of HGA sharply increased within the first 24 h. This coincides with the results of Gonzales-Medina et al. [19]. The concomitant decrease in HGB concentration led to the assumption that HGA was released from HGB. Hypoglycin B is a dipeptide of HGA and glutamic acid. Its hydrolysis liberates HGA, and HGA concentration increases. On the basis of the measured concentrations during the incubation period, we modeled a rapid conversion of HGB to HGA, which was accompanied by a progressive decrease in the HGA concentration. The maximal HGA concentration was predicted to be reached at 13.7 h.

In the incubations of SMS made with autoclaved (inactive) ruminal fluid, a similar conversion of HGB to HGA was observed (Figure 2F), although this was not as pronounced or complete as in the incubations with active ruminal fluid. The conversion of HGB to HGA in both types of incubations points to a process independent from ruminal microbial assistance. In ackee fruits, HGA is primarily located in the arilli and seeds, but HGB exclusively in the seeds [17]. Ripening decreases HGA in the arilli [17]. In the seeds, HGA is converted to HGB by γ-glutamyl-transpeptidase [17]. Similar processes could be applicable to SMS and sycamore maple seedlings [27]. For sycamore maple, El-Khatib et al. [16] have shown that HGA and HGB occur together in the leaves and seeds, although the proportion of HGB is far higher in the seeds. Gonzales-Medina et al. [28] reported that mowing significantly increased HGA concentration in seedlings, and hypothesized that stress upregulates HGA synthesis. Plants are probably able to release HGA if required (in a stressful situation), and the HGA-HGB conversion seems to be a bidirectional process. Generally, toxins are synthesized by plants as defensive agents against herbivory, pathogens, or environmental stressors [29]. From other plants, it is known that they protect themselves against herbivores through a combination of a (pro)toxin with an enzyme that liberates the actual toxin upon damage of the plant, e.g., by grazing [30]. This mechanism seems a likely explanation for the observed effects in maples.

In the rumen environment, the release of HGA from HGB, and also the possible release of MCPrG from γ-glutamyl-MCPrG in the maple seeds, could be induced by one or several plant enzymes that are released from their cellular compartments by the mechanical stress of the grinding of the seeds. Additionally, the cleavage of HGB could also be mediated by microbial enzymes present in ruminal fluid, which need to be further characterized. We did not detect any of the investigated metabolites of HGA or MCPrG (MCPA and MCPF conjugates with glycine and carnitine) after incubation in ruminal fluid batch cultures. The investigated MCPA and MCPF conjugates were found in the urine and milk of dairy cows, and in the serum of ewes [19,20], whereas Krägeloh et al. [31] did not find any metabolites in the feces of horses. These results indicate that metabolization and clearance mainly occur outside the gastrointestinal lumen, probably in the liver. However, the transamination of HGA to MCP-pyruvate and of MCPrG to MCP-glyoxalate could occur in the rumen as in other parts of the digestive system [32]. Although the current in vitro study results can only be transferred to the in vivo situation to a limited extent, it seems that the reduced susceptibility to HGA and MCPrG intoxication, described by Renaud et al. [10] and Engel et al. [20] in sheep and cows after ingestion of sycamore seeds and seedlings, is not primarily related to microbial transformation of the toxins in the rumen. However, it should be noted that the adaptation of ruminal microorganisms to toxin exposure does not occur in the batch culture system. To investigate this, semi-continuous systems such as RUSITEC or in vivo studies would have to be used.

### 2.2. Gas Production, pH, Redox Potential, and Microbial Metabolites

The gas production kinetics in the batch cultures are summarized in Table 1. The Gompertz non-linear regression function generally fitted gas production data (*R*² > 0.99). Asymptotic gas production ranged from 200 mL/g dry matter (DM) in the cellulose–starch mixture (CSM) to 453 mL/g DM in SMS, and differed among the variants (*p* < 0.05). Gas production was distinctly delayed in SMS incubations compared to those with CSM and PCM (*p* < 0.05), which is why false extrapolation could possibly have led to an overestimation of the gas production asymptote. As in the SMS incubations, one-third of the entire substrate was the milled seeds; nevertheless, they were a relevant source of energy and nutrients. The analyzed nutrient composition of the seeds is summarized in Table S1. The seeds contained 227 g cellulose, 80 g hemicellulose, and 223 g non-fiber carbohydrates/kg DM. The degree of lignification was 0.36 and the gross energy content was 18.4 MJ/kg DM. The high content of structural carbohydrates, but primarily the quantity effect, led to the higher and delayed gas production.

The inoculum pH was 7.2 ± 0.46 before the inoculation of the substrates and around 7.0 at the beginning of the incubation period (0 h). The pH then decreased to a minimum of 6.6 (*p* < 0.001; Table 2). Differences among the incubated variants were not found. The redox potential was around −180 mV at 0 h (Table 2). The redox potential decreased up until 48 h, and was then lowest in SMS (−280 mV) (*p* < 0.01). Again, no differences were found among the variants. The redox potentials around or lower than −180 mV and the pH values in the range from 6.6 to 7.0 indicated a stable environment in the batch cultures, regardless of the incubated variant. This is a prerequisite for the simulation of ruminal fermentation processes. Müller and Kirchner [33] reported that until up to 30 min of incubation, remaining oxygen from feed and water is consumed by facultative aerobic microorganisms, and this results in a general decrease in the redox potential. After 48 h, the redox potential was lowest in SMS. Recent studies have reported the high reductive ability of SMS due to a high NADPH/NADP+ ratio [34,35]. In the inoculum made with autoclaved ruminal fluid (0 h), the pH was 7.1 ± 0.26 and the redox potential was −1.3 ± 28 mV. After a 48 h incubation period, the pH was 7.9 ± 0.53 in PCM and 7.9 ± 0.46 in SMS incubations. The redox potential was −226 ± 74.3 mV in PCM and −306 ± 40.1 mV in SMS incubations.

In the batch cultures, mainly acetic acid, propionic acid, and *n*-butyric acid were produced (Table 2). Only very low concentrations of iso-butyric acid, *n*-valeric acid, and iso-valeric acid have been detected. *n*-Caproic acid was not found. Lactic acid was not measured. The SCFAs accumulated in the liquid phase of the fermenters during the incubation time course (Table 2). An exception was the decrease in the concentration of iso-butyric acid and iso-valeric acid in SMS incubations (*p* < 0.001). After 48 h of incubation, acetic acid was elevated in SMS compared to the other variants (*p* < 0.001). In contrast, propionic acid, iso-butyric acid, and iso-valeric acid were lower (*p* < 0.05). In CSM, PCM, and SMS, the ratio of acetic acid to propionic acid was 3.8, 5.0, and 5.0 at 0 h and 1.7, 2.0, and 5.6 at 48 h, respectively (Table 2). The acetic acid to propionic acid ratio increased in SMS after 48 h (*p* < 0.05). Microbial degradation of rapidly fermentable carbohydrates usually leads to more propionic acid than acetic acid [36,37]. The opposite can be observed if slowly fermentable carbohydrates prevail. The decrease in the proportion of iso-SCFAs is also congruent with the growth of cellulolytic bacteria [38]. The NH_3_ concentration of ruminal fluid was 10 ± 0.61 mmoL/L. The inoculum contained 11 ± 0.81 mmol/L NH_3_, of which a part referred to the buffer solution. On no account, a net increase in NH_3_ from substrate fermentation has been detected (Table 2). The results indicate that SMS was used as an additional substrate by the batch culture microorganisms. However, the presence of toxins seemed not to relevantly affect the microbes or their environment.

**Table 1:** Gas production kinetics estimated from incubation of a cellulose–starch mixture (CSM) as control, CSM and a pure HGA and MCPrG mixture (PCM), and CSM and milled sycamore maple seeds (SMS) in a ruminal fluid batch culture.

Item	*a*	*b*	*c*	*b* + *c*	RMSE	*R* ^2^
CSM	200 (196 205)	18.6 (18.2 18.9)	8.5 (8.0 9.0)	27.1	6.42	0.993
PCM	272 (269 275)	17.5 (17.3 17.7)	10.7 (10.4 11.0)	28.2	3.22	0.999
SMS	453 (375 581)	43.5 (37.8 51.6)	32.1 (28.1 37.4)	75.6	5.51	0.990

Model parameters: *a*, asymptotic maximal gas production (mL/g dry matter); *b*, time (h) until which one-third of *a* is produced; and *c*, time (h) between *b* and *b* + *c*, the time (h) until which 70% of *a* is produced. 95% confidence intervals are given in brackets. Gas production was modeled using Gompertz non-linear regression function according to Dhanoa et al. [39]. Abbreviations: RMSE, root mean square error.

**Table 2:** Least squares means and standard errors of pH, redox potential, the concentration of short chain fatty acids (SCFAs), and NH_3_ measured in the liquid phase of the fermenters at 0 and 48 h of incubation of a cellulose–starch mixture (CSM) as control, CSM and a pure HGA and MCPrG mixture (PCM), and CSM and milled sycamore maple seeds (SMS) in a ruminal fluid batch culture.

	CSM		PCM		SMS	
Analyte	0 h	48 h	0 h	48 h	0 h	48 h
pH	7.0 (0.14)	6.8 (0.14)	6.9 (0.024) ^a^	6.6 (0.024) ^b^	6.9 (0.051) ^a^	6.7 (0.051) ^b^
Redox potential	−178 (34.2)	−215 (34.2)	−189 (22.5)	−249 (22.5)	−184 (16.6) ^b^	−280 (16.6) ^a^
Acetic acid	18 (8.5)	41 (8.5) ^AB^	22 (1.2) ^b^	48 (1.2) ^aB^	22 (2.8) ^b^	64 (2.8) ^aA^
Propionic acid	6.8 (2.7) ^b^	22 (2.7) ^aA^	4.3 (0.88) ^b^	24 (0.88) ^aA^	4.5 (0.61) ^b^	11 (0.61) ^aB^
C_2_:C_3_ ratio	3.8 (0.82)	1.7 (0.82) ^B^	5.0 (0.094) ^a^	2.0 (0.094) ^bB^	5.0 (0.16)	5.6 (0.16) ^A^
*n*-Butyric acid	1.7 (1.1)	4.5 (1.1)	2.1 (0.50) ^b^	4.9 (0.50) ^a^	2.9 (0.55) ^b^	6.3 (0.55) ^a^
iso-Butyric acid	0.42 (0.18)	0.75 (0.18) ^A^	0.20 (0.046) ^b^	0.60 (0.046) ^aA^	0.21 (0.013) ^a^	0.10 (0.013) ^bB^
*n*-Valeric acid	0.14 (0.061)	0.35 (0.061)	0.14 (0.023) ^b^	0.38 (0.023) ^a^	0.15 (0.030) ^b^	0.39 (0.030) ^a^
iso-Valeric acid	0.58 (0.23)	1.1 (0.23) ^A^	0.30 (0.069) ^b^	0.91 (0.069) ^aA^	0.32 (0.017) ^a^	0.14 (0.017) ^bB^
NH_3_	−1.2 (1.2)	−5.7 (1.2)	−1.8 (0.41) ^a^	−5.3 (0.41) ^b^	−1.3 (0.39) ^a^	−4.8 (0.39) ^b^

The redox potential is given as mV; the concentration of SCFAs as mmol/L; NH_3_ is expressed as the difference between concentrations (mmol/L) in test fermenters and background fermentation in blanks. Standard errors are given in brackets. ^a,b^ Lower-case superscripts indicate differences among incubation time points; ^A,B^ upper-case superscripts indicate differences among the incubated variants (*p* < 0.05).

### 2.3. Correlations

A correlation analysis was performed using the pH and redox potential, as well as HGA, HGB, MCPrG, SCFAs, and NH_3_ measured at 24 h of the incubation period. In PCM incubations, negative correlations were detected between HGA or MCPrG and acetic acid (*p* < 0.01), propionic acid (*p* < 0.001), *n*-butyric acid (*p* < 0.01), and iso-butyric acid (*p* < 0.01) (Table 3). This simply results from rapidly decreasing concentrations of the toxins and simultaneously accumulating SCFAs. In SMS incubations, HGA was positively correlated with acetic acid, propionic acid, and *n*-valeric acid concentrations (*p* < 0.01) (Table 3). Coherently, negative correlations were found between HGB and these SCFAs (*p* < 0.05) (Table 3). In SMS incubations, HGA and MCPrG concentrations initially increased, while HGB decreased, and SCFAs accumulated. The correlation analysis did not reveal any effect of toxins on microbial activity in the batch cultures.

In PCM incubations, a strong linear correlation was found between HGA and NH_3_ (*r* = 0.95; *p* < 0.001) and MCPrG and NH_3_ (*r* = 0.96; *p* < 0.001). Note, NH_3_ is given as the difference between its concentration in the test fermenters and its concentration in the blank fermenters. Thus, the more negative these values are, the less N is present after 24 h as NH_3_. This is an indication that any N available from the toxins is incorporated into microbial protein.

## 3. Conclusions

The current study revealed a release of HGA from HGB in SMS incubated in ruminal fluid batch cultures and a similar release of MCPrG from γ-glutamyl-MCPrG. Simultaneously, HGA and MCPrG concentrations decreased during the 48 h incubation period. In the incubations with the pure substances, HGA and MCPrG were rapidly and completely transformed. Possible explanations for the decrease in HGA and MCPrG concentrations could be microbial protein synthesis or conversion, chemical transformation, or binding to ruminal fluid constituents such as fiber and proteins. The HGB to HGA conversion could be catalyzed by plant enzymes present in the seeds and released by grinding. The conversion of HGB to HGA and the transformation of HGA and MCPrG, to a lesser extent, also occurred in incubations with autoclaved ruminal fluid. This points to a process not solely related to enzymes. Metabolites of HGA or MCPrG (MCPA and MCPF conjugates) were not detected. Moreover, there was no evidence that the presence of the toxins affected the rumen environment or microbial metabolites.

## 4. Materials and Methods

### 4.1. Donor Animals

For the current in vitro experiment, ruminal fluid was obtained from two adult rumen-cannulated wethers (*Ovis aries*). The housing and feeding of the sheep were described in detail by Bachmann et al. [40]. The sheep were housed in an open barn and received meadow hay ad libitum. Additionally, each animal was offered 200 g concentrate, pelleted to Ø 3 mm (IBEKA PANTO Schäferstolz; HL Hamburger Leistungsfutter GmbH, Hamburg, Germany) and 10 g mineral feed (basu-kraft^®^ Top-Mineral; BASU Heimtierspezialitäten GmbH, Bad Sulza, Germany) per day. The sheep had the opportunity to ingest straw from the bedding and had free access to tap water.

Ruminal fluid was sampled before feeding in the morning, and approximately 1.5 h prior to the start of an in vitro run. Immediately after removal from the rumen, it was roughly filtered through a one-layered cheesecloth. Ruminal fluid from the two wethers was mixed in equal shares. The ruminal fluid had a temperature of 34 ± 3.0 °C when it arrived in the laboratory.

### 4.2. Substances and Preparations

(S)-HGA (purity 85%), HGB (γ-glutamyl-hypoglycin; purity 98%), α-MCPrG (purity 97%), and MCPA-carnitine (purity 97%) standards were purchased from Toronto Research Chemicals Inc. (Toronto, ON, Canada). MCPA-glycine (purity 97%) and MCPF-glycine (purity 97%) standards were purchased from IsoSciences LLC (Ambler, PA, USA).

Stock HGA, MCPrG, MCPA-glycine, MCPF-glycine, and MCPA-carnitine standard solutions (0.1 mg/mL) were prepared in 50% acetonitrile in water (*v*/*v*). A working standard mixture (1.0 µg/mL) was prepared by mixing the stock solutions and diluting them with 5% MeOH in water (*v*/*v*). This was used as the calibration mixture. For calibration, a serial dilution of the mixture was prepared using 5% MeOH and blank inactivated ruminal fluid to obtain 0.5, 1.0, 2.5, 4.0, 5.0, 10, 25, 50, 100, and 150 ng/mL (matrix-matched calibration). Due to the possible hydrolysis of HGB into HGA, a separate series of calibration solutions was prepared for HGB.

For the incubation in batch cultures, a solution with 500 ng HGA/MCPrG mixture/mL was prepared.

*Acer pseudoplatanus* seeds were collected in fall 2020 from a pasture north of Berlin, Germany, air-dried, and stored at room temperature. The seeds were then milled in a ball mill (MM400; Retsch, Haan, Germany) using a 50 mL stainless steel beaker and a 20 mm steel ball at a frequency of 25/s for 45 s.

Purified cellulose (Vitacel^®^ R200; J. Rettenmaier and Söhne GmbH & Co. KG, Rösenberg, Germany) and corn starch were mixed in a ratio of 80:20 (*w*/*w*) and used as the basic substrate (CSM) to feed the batch cultures in the in vitro trials. Both substrates are routinely used in the laboratory for feed evaluation purposes, as a reference for marking the upper and lower borders of gas production obtained from the inoculation of the majority of feeds.

### 4.3. Batch Culture Incubation

For the in vitro incubations, the ANKOM RF Gas Production System (ANKOM Technology, Macedon, NY, USA) was used. The inoculum was prepared in a 1:2 ratio (*v*/*v*) of ruminal fluid and buffer solution under carbon dioxide flush, following the protocol of the Association of German Agricultural Inspection and Research Institutes (VDLUFA) (method no. 25.1) [41]. The buffer solution contained 474 mL water, 0.12 mL trace element solution (13.2 g CaCl_2_ × H_2_O, 10.0 g MnCl_2_ × 4 H_2_O, 1.0 g CoCl_2_ × 6 H_2_O, and 8.0 FeCl_3_ × 6 H_2_O in 100 mL water), 237 mL buffer solution (33.0 g NaHCO_3_ and 6.0 g NH_4_HCO_3_ in 1 L water), 237 mL macronutrient solution (5.7 g Na_2_HPO_4_, 6.2 g KH_2_PO_4_, and 0.6 g MgSO_4_ × 7 H_2_O in 1 L water), 1.22 mL resazurin solution (100 mg resazurin, sodium salt in 100 mL water), and reduction solution (2.0 mL NaOH, 1 mol/L, 285 mg Na_2_S × 7 H_2_O in 47.5 mL water) [41,42].

Four consecutive runs (four replicates) were performed. In each run, the following variants were incubated in duplicate for five sampling time points (i.e., 2 × 5 fermenters/variant): CSM, CSM plus HGA/MCPrG mixture (PCM), and CSM plus SMS. Two fermenters of each variant were removed at 0, 1, 2, 4, 8, 24, and 48 h, respectively, and sampled. Additionally, four blank fermenters (only ruminal fluid and buffer solution) were applied within each run, as well as two fermenters containing only CSM, for the 0 and 48 h sampling time points. In order to distinguish between non-fermentation and fermentation reactions [43,44], controls were included, with CSM, PCM, and SMS, and autoclaved ruminal fluid (121 °C for 15 min), and sampled at 0 and 48 h time points. Each fermenter contained 30 mL inoculum, and if applicable, either 200 mg CSM, CSM and 1.5 mL PCM (i.e., 500 ng/mL HGA/MCPrG mixture), or CSM and 100 mg SMS. The fermenters were glass bottles with 136 ± 2.68 mL actual volume (i.e., 106 mL headspace volume) and each was capped with a gas pressure measuring module. The fermenters were randomly distributed to three shaking water baths (SW23; JULABO GmbH, Seelbach, Germany) set to 80 r/min agitation and 39 °C, with automatic temperature control.

The pH and redox potential of the original ruminal fluid and the autoclaved ruminal fluid were measured prior to each run, as well as after incubation at each sampling time point, using InLab Expert Pro and InLab Redox electrodes (Mettler Toledo GmbH, Vienna, Austria). The pH of the original ruminal fluid immediately prior to the preparation of the inoculum was 6.6 ± 0.12 and the redox potential −207 ± 44.1 mV. In the autoclaved ruminal fluid, a pH of 6.9 ± 0.44 and a redox potential of −43.8 ± 28.8 mV were measured.

Cumulative gas pressure was measured in real time and recorded in a 5 min interval. A threshold of 1.5 psi was chosen for the automatic release of accumulated gasses. This prevents the supersaturation of fermentation gas in the medium under high gas pressure [45]. To purge oxygen out of the bottles before data acquisition, each bottle was vented with argon through the modules’ Luer port until the inner pressure exceeded 8 psi. The valve open time was set to 150 ms. Cumulative gas pressures were applied to blank correction using the mean gas pressure of the blanks and converted to mL of gas by applying the Ideal Gas Law and Avogadro’s Law.

### 4.4. Sampling

At the sampling time points defined above, 15 mL of the liquid phase from each fermenter was obtained by pipetting, immediately flushed with liquid nitrogen to prevent further enzymatic activity, and stored at −80 °C until analysis of toxins and their metabolites. Additionally, and coincidentally in time, 6 mL was taken for analysis of SCFAs and NH_3_, and stored at −20 °C.

### 4.5. Chemical Analyses

All analytical solvents used in this work were at least analytical grade. The solvents used for LC-MS/MS were of LC-MS grade.

For the analysis of maple toxins, samples were vortexed, and 2 mL aliquots were centrifuged for 5 min at 14,000 rpm (18,407× *g*) and 20 °C (Centrifuge 5424 R; Eppendorf, SE, Hamburg, Germany). From the supernatant, 100 µL was diluted with 900 µL 5% MeOH in water and filtered using a 0.2 µM centrifugal filter (modified nylon) into vials and analyzed by LC-MS/MS. LC-MS/MS was performed in multiple reaction monitoring (MRM) modes using an Agilent 1290 Infinity II UPLC system (Agilent Technologies, Waldbronn, Germany) coupled to a Q-Trap 6500+ mass spectrometer, equipped with an IonDrive™ Turbo V electrospray ionization (ESI) source (AB Sciex Germany GmbH, Darmstadt, Germany). Chromatographic reversed-phase (RP) separation with 10 μL injection volume was achieved on a Waters Acquity UPLC BEH C18 column (150 × 2.1 mm, 1.7 μm particle size; Waters GmbH, Eschborn, Germany) at a flow rate of 0.3 mL/min and a column oven temperature of 40 °C. The binary mobile phase consisted of 5 mM ammonium formate and 0.1% formic acid in water (eluent A) and methanol (eluent B). The gradient elution was adopted as follows: 0 min 0% B; 1 min 0% B; 2 min 50% B; 3 min 70% B; 5 min 100% B; 7 min 100% B; 7.5 min 0% B; and 10 min 0% B. The detection was conducted using positive ionization mode. The MRM transitions and MS/MS conditions are summarized in Table S2. The quantification of HGA, HGB, MCPrG, and the metabolites was performed using external calibration with matrix-matched calibration standards.

Microbial metabolites were analyzed in the liquid phase of the fermenters. For the analysis of acetic acid, propionic acid, *n*-butyric acid, iso-butyric acid, *n*-valeric acid, iso-valeric acid, and *n*-caproic acid, 2 mL of sampled fluid was centrifuged for 5 min at 2000× *g*. Afterwards, 1 mL of the supernatant was transferred to a new vessel and mixed with 100 µL of internal standard consisting of 1.5 g 4-methyl-valeric acid (iso-caproic acid) dissolved in 100 mL of 80% formic acid. The samples were mixed and centrifuged a second time for 3 min at 2000× *g*. The clear supernatant was then transferred to vials and measured by gas chromatography. Gas chromatography was performed using a Shimadzu GC2010 (Shimadzu Corp., Kyoto, Japan) fitted with a flame ionization detector. Analytes were separated on an SGE BP21 column (30 m × 0.53 mm × 0.5 µm; Trajan Scientific and Medical, Ringwood, Australia) following on-column injection. The following settings were used: 0.5 µL injection volume, 180 °C injection temperature, a constant pressure of 22.7 kPa (i.e., 29.7 cm/s linear velocity and 3.64 mL/min column flow), 85 °C initial oven temperature, which was raised up by 8 °C/min to 200 °C and held for 6 min, and 200 °C detection temperature. Helium was used as a carrier and make-up gas. The concentration of the target analytes was determined on the basis of a 6-point external standard calibration. The concentration of NH_3_ was determined according to Voigt and Steger [46]. Briefly, 4 mL of boric acid solution (5 g boric acid dissolved in 200 mL alcohol mixed with 10 mL indicator solution, filled up to 1 L with distilled water) was transferred to the Erlenmeyer flask of a micro diffusion vessel. Subsequently, 1 mL of inoculum was undercoated with 1 mL of potassium carbonate solution in the container at the ground glass stopper. The mixture was then incubated for 24 h and the change in color was subsequently back-titrated using 0.01 N HCl solution.

The maple seeds were analyzed in terms of DM (method no. 3.1), crude protein (method no. 4.1.1), acid ether extract (method no. 5.1.1 B), crude fiber (method no. 6.1.1), neutral detergent fiber (method no. 6.5.1), acid detergent fiber (method no. 6.5.2), acid detergent lignin (method no. 6.5.3), and ash (method no. 8.1) [41]. Neutral detergent fiber was determined after treatment with heat-stable amylase, which was added to the neutral detergent solution. Neutral detergent fiber and acid detergent fiber were expressed exclusive of residual ash. Starch was determined using the amyloglucosidase method (method no. 7.2.5) [41]. The gross energy concentration was determined by bomb calorimetry using a C7000 Oxygen Bomb Calorimeter (IKA Werke, Staufen, Germany). Macro and trace elements were analyzed by inductively coupled plasma optical emission spectrometry (Varian 715-ES ICP-OES; Agilent Technologies Inc., Santa Clara, CA, USA) following extraction, as described by Rodehutscord and Dieckmann [47]. The seed proteins were hydrolyzed with hydrochloric acid and individual amino acids were analyzed using a Biochrom 30 Amino Acid Analyser fitted with a PEEK-Sodium Prewash column (100 mm × 4.6 mm) and a PEEK-Oxidised Feedstuff column (200 mm × 4.6 mm; Biochrom Ltd., Cambridge, UK) (method no. 4.11.6) [41].

### 4.6. Statistical Analysis

Statistical analysis was performed using the SAS 9.4 software package (SAS Institute Inc., Cary, NC, USA).

The conversion from HGB to HGA and the associated change in the concentration of the two substances was modeled using the following differential equation system:

$$y_{HGB}^{\prime }(t)={-k\cdot y}_{HGB}(t)$$

$$y_{HGA}^{\prime }(t)={k\cdot y}_{HGB}(t)-l{\cdot y}_{HGA}(t)$$

The first differential equation models the change (reduction) in HGB concentration proportional to the existing HGB concentration for each time point. Since the HGB concentration decreases monotonically as a function of time, the sign of the proportionality factor (rate constant) *k* (*k* > 0) is negative. The second differential equation models the change in HGA concentration. In addition to the expected reduction in the concentration of HGA (term $-l\cdot y_{HGA}$), with the rate constant *l*, this equation also considers that the HGB concentration change takes place by converting HGB into HGA (term $+k\cdot y_{HGB}$). Since the values at time *t* = 0 for HGA (*HGA*_0_ = $y_{HGA}$ (*t* = 0)) and HGB (*HGB*_0_ = $y_{HGB}$ (*t* = 0)) can be measured, this results in a so-called initial value problem, whose analytical solution can be given directly as follows (l≠k):

$$y_{HGB}(t)={HGB}_{0}\cdot e^{-k\cdot t}$$

$$y_{HGA}\left(t\right)=\frac{k}{l-k}{HGB}_{0}\cdot e^{-k\cdot t}+\left({HGA}_{0}-\frac{k}{l-k}{HGB}_{0}\right)\cdot e^{-l\cdot t}$$

The model parameters *HGA*_0_, *HGB*_0_, *k*, and *l* can be estimated using non-linear regression with the MODEL procedure, e.g., using all individual measurements. Alternatively, the given initial value problem can be solved using the same procedure if the HGA and HGB mean values for the different time points are used.

The recorded gas production data set was scaled down to a 0.5 h resolution, and non-linear regression analysis was performed using the MODEL procedure and Gompertz function [39]. Differences in the estimated parameters of gas production kinetics were assessed using 95% confidence intervals.

Least squares means were estimated for pH, redox potential, SCFAs, and NH_3_ using the MIXED procedure and the following model: *y_ij_* = *µ* + *α_i_* + *β_j_* + *αβ_ij_* + *ε_ij_*, where *y_ij_* is the analyte, *µ* is the general mean, *α_i_* is the fixed effect of incubation time *i* (*i* = 1, …, 48 h), *β_j_* is the fixed effect of the variant *j* (*j* = 1, …, 3; 1 = CSM, 2 = PCM, 3 = SMS), *αβ_ij_* is the interaction of *α_i_* and *β_j_*, and *ε_ij_* is the random residual effect with *ε_ij_*~N (0, σ^2^*ε_j_*).

Correlation coefficients between toxins, fermentation parameters, and microbial metabolites, were computed using the CORR procedure. As the levels of maple toxins at time points beyond 24 h were sometimes below the measurement range, the 24 h time point was used for the correlation analysis.

Gaussian distribution of the studentized residuals was confirmed using the UNIVARIATE procedure. A *p* < 0.05 was considered indicative of significant differences.

## Figures and Tables

**Table 3:** Pearson, Spearman, and Kendall’s Τ correlation coefficients among HGA, HGB, and MCPrG concentrations and pH, redox potential, the concentration of short chain fatty acids, and NH_3_ in the liquid phase of the fermenters at 24 h of incubation of a cellulose–starch mixture (CSM), a pure HGA and MCPrG mixture (PCM), and CSM and milled sycamore maple seeds (SMS) in a ruminal fluid batch culture.

	Pearson					Spearman					Kendall’s Τ				
	PCM		SMS			PCM		SMS			PCM		SMS		
	HGA	MCPrG	HGA	HGB	MCPrG	HGA	MCPrG	HGA	HGB	MCPrG	HGA	MCPrG	HGA	HGB	MCPrG
pH	−0.32 *	−0.22	0.17	0.04	0.12	−0.14	−0.12	0.27	0.06	0.16	−0.19	−0.15	0.18	0.06	0.11
Redox potential	0.14	0.20	−0.22	0.28	0.04	0.29 *	0.32 *	−0.27	0.36 *	0.02	0.20 *	0.22 *	−0.18	0.24 *	0.03
Acetic acid	−0.86 ***	−0.88 ***	0.72 **	−0.84 ***	−0.27	−0.77 ***	−0.84 ***	0.77 ***	−0.72 **	−0.28	−0.58 **	−0.67 ***	0.55 **	−0.50 **	−0.18
Propionic acid	−0.95 ***	−0.96 ***	0.42	−0.56 *	−0.23	−0.83 ***	−0.86 ***	0.81 ***	−0.69 **	−0.23	−0.67 ***	−0.70 ***	0.60 **	−0.45 *	−0.13
*n*-Butyric acid	−0.70 **	−0.71 **	0.24	−0.16	−0.07	−0.65 **	−0.74 **	0.47	−0.22	−0.05	−0.51 **	−0.61 **	0.35	−0.13	−0.02
iso-Butyric acid	−0.63 **	−0.64 **	−0.43	0.43	−0.06	−0.73 **	−0.66 **	−0.44	0.31	−0.02	−0.55 **	−0.52 **	−0.29	0.27	−0.03
*n*-Valeric acid	−0.12	−0.14	0.76 ***	−0.82 ***	−0.21	−0.11	−0.21	0.79 ***	−0.81 ***	−0.24	−0.09	−0.17	0.58 **	−0.63 **	−0.19
iso-Valeric acid	0.37	0.38	−0.43	0.55 *	0.19	0.24	0.32	−0.49	0.40	0.15	0.17	0.25	−0.34	0.30	0.10
NH_3_	0.95 ***	0.96 ***	−0.40	0.37	0.58*	0.80 ***	0.76 ***	−0.47	0.40	0.52*	0.60 **	0.55 **	−0.32	0.32	0.37 *

Abbreviations: HGA, hypoglycin A; HGB, hypoglycin B; MCPrG, methylene cyclopropyl glycine. Asterisks indicate significant correlations: * *p* < 0.05, ** *p* < 0.01, and *** *p* < 0.001.

## Data Availability

The raw data supporting the conclusions of this article will be made available by the authors on request.

## Supplementary Materials

The following supporting information can be downloaded at: https://www.mdpi.com/article/10.3390/toxins17020046/s1. Table S1: analyzed energy and nutrient composition of sycamore maple seeds; Table S2: mass transitions and conditions for LC-MS/MS determination of HGA, HGB, MCPrG, γ-glutamyl-MCPrG, MCPA-glycine, MCPF-glycine, and MCPA-carnitine in ruminal fluid.
