# Supplementary material for: Release of Hypoglycin A from Hypoglycin B and Decrease of Hypoglycin A and Methylene Cyclopropyl Glycine Concentrations in Ruminal Fluid Batch Cultures
Source: Toxins (Basel). 2025 Jan 21;17(2):46. doi: 10.3390/toxins17020046 (PMC11860860; doi:10.3390/toxins17020046)
Supplement: Supplementary file 1 [file toxins-17-00046-s001.zip › Table-S1.pdf]

Table S1. Analyzed energy and nutrient composition of sycamore maple seeds

Dry matter	920
Crude ash	97
Crude protein	179
Acid ether extract	69
Crude fiber	254
aNDFom	432
ADFom	353
ADL	126
Gross energy	19.9
Cysteine	5.1
Methionine	3.1
Aspartic acid	13.6
Threonine	6.3
Serine	8.5
Glutamic acid	41.8
Proline	7.1
Glycine	7.7
Alanine	5.9
Valine	6.6
Isoleucine	6.9
Leucine	11.1
Phenylalanine	6.1
Lysine	7.8
Arginine	12.7
Tryptophan	1.8
P	4.5
Ca	18.8
K	22.9
Na	0.11
Mg	1.7
Zn	44.0
Mn	33.0
Cu	10.5
Fe	108.1

Dry matter is given as g/kg, crude nutrients, detergent fibers, amino acids, P, Ca, K, Na, and Mg are given as g/kg dry matter, Zn, Mn, Cu, and Fe are given as mg/kg dry matter, and gross energy is given as MJ/kg dry matter.
Abbreviations: ADFom, acid detergent fiber; ADL, acid detergent lignin; aNDFom, neutral detergent fiber.
aNDFom was treated with amylase; aNDFom and ADFom are expressed exclusive of residual ash.
