# Supplementary material for: Release of Hypoglycin A from Hypoglycin B and Decrease of Hypoglycin A and Methylene Cyclopropyl Glycine Concentrations in Ruminal Fluid Batch Cultures
Source: Toxins (Basel). 2025 Jan 21;17(2):46. doi: 10.3390/toxins17020046 (PMC11860860; doi:10.3390/toxins17020046)
Supplement: Supplementary file 1 [file toxins-17-00046-s001.zip › Table-S2-new.pdf]

Table S2. Mass transitions and conditions for LC-MS/MS determination of HGA, HGB, MCPrG, γ-glutamyl-MCPrG, MCPA-glycine, MCPF-glycine, and MCPA-carnitine in ruminal fluid

Analyte	Precursor ion (m/z)	DP (V)	EP (V)	Product ions (m/z)	CE (V)	CXP (V)	Expected RT (min)
MCPrG	128.0	35.8	4.45	64.8	25.8	7.0	1.75
				92.0	20.5	14.0	
γ-glutamyl-MCPrG	257.1	31.0	10.0	128.1	19.1	17.0	3.42
				84.0	32.0	13.9	
HGA	142.0	15.0	2.60	73.9	11.0	8.0	3.11
				46.2	18.5	9.0	
HGB	271.1	31.0	10.0	74.1	21.3	9.9	3.75
				142.1	19.1	17.0	
MCPF-glycine	156.1	35.0	7.23	80.9	15.0	9.5	3.46
				53.0	29.3	6.4	
MCPA-glycine	170.1	33.0	11.50	73.8	19.4	8.2	3.84
				68.9	15.6	7.5	
MCPA-carnitine	256.2	32.0	13.50	84.9	27.0	13.5	3.67
				197.1	20.0	18.5	

For all analytes, the dwell time was 80 ms. Abbreviations: CE, collision energy; CXP, cell exit potential; DP, declustering potential; EP, entrance potential; HGA, hypoglycin A; HGB, hypoglycin B; LC, liquid chromatography; MCPA, methylene cyclopropyl acetyl; MCPF, methylene cyclopropyl formyl; MCPrG, methylene cyclopropyl glycine; MS/MS, tandem mass spectrometry; RT, retention time.
